# Targeted imaging: the key for direct visualization of myocardial inflammation in patients?

**DOI:** 10.1007/s12350-023-03370-9

**Published:** 2023-09-20

**Authors:** Theresa Reiter

**Affiliations:** Department of Internal Medicine I, Cardiology, University Hospital Wuerzburg, Wuerzburg, Germany

Acute ischemia has an immense impact on the cellular and structural integrity of the myocardium. It triggers an inflammatory response that is a vital factor for the further clinical and prognostic outcome.^1^ As such, inflammation serves as promising target for individualized therapeutic interventions. Already, a first human study has used antibodies to modulate specific signaling pathways of the innate immune system in the CANTOS trial.^2,3^ The results showed a reduction in the primary endpoints, including non-fatal or fatal myocardial infarction and stroke. However, the results also demark the challenge to identify the patients who benefit the most from a therapy connected with high costs and risks due to manufactured immunosuppression.^4^

Therapeutic approach like CANTOS make use of knowledge obtained from in vitro and animal experiments. These experiments demonstrate the impact of macrophages and lymphocytes on myocardial healing and remodeling and also play a role in the development of complications such as an increasing incidence of ventricular thrombi when the monocyte/macrophage function is impaired.^5^

However, the transfer from bench to bedside remains a challenge. In patients with acute myocardial infarction, the clinical presentation is by far more complex and variable than a precisely defined experiment can reproduce. Histological prove of myocardial cell infiltration is hardly obtainable and quantification of the inflammatory process itself is limited to parameters derived from peripheral blood samples.^6^

Established clinical imaging methods allow a detailed analysis of the immediate and long-term effects of inflammation on the myocardium. Echocardiography is a readily available and fast tool for detection of myocardial function, wall motion, and pericardial effusion. It is a valuable tool for early risk stratification after myocardial infarction and offers profound insight into both systolic and diastolic functions.^7^ Cardiac MRI adds the unique information of changes in the extracellular volume such as edema and necrotic tissue in the acute setting. In the chronic stage after myocardial infarction, it detects the scar tissue as result of the post-infarction inflammation and remodeling. Thus, it is used in clinical trials for the outcome assessment.^8^

However, these imaging techniques examine the effects of inflammation on the myocardial damage, but do not elucidate the process of inflammation itself. For this purpose, nuclear medicine is the imaging modality to turn to.

Among the tracers used routinely for oncologic indications, SSTR2-targeted tracers has proven its potential for translation into detection of inflammation. SSTR2-targeted PET is used to detect neuroendocrine tumors that show a significant upregulation of SSTR2 expression. This is useful not only for the detection and monitoring of tumor disease but also for therapeutic interventions.^9^

Besides neuroendocrine tumorous tissues, a variety of cell groups expresses SSTR2. Among these, macrophages are especially noteworthy as they are key to open the door of translation. PET has a whole body or at least thoracic read out, and as such, offers information on other not primarily targeted tissues and organs. Earlier works successfully used SSTR2-targeted imaging in oncologic patients in order to detect inflammation in atherosclerotic plaques of larger arteries and to connect the imaging findings to cardiovascular risk factors.^10^ Besides detection of SSTR2-targeted tracer uptake in large vessels, other works were able to discriminate between high-risk and low-risk lesions in carotid and coronary vessels.^11^

From these large-vessel analyses, the next step toward for direct visualization of myocardial inflammation was pursued in smaller pilot series and studies that focused on myocardial inflammation either with inflammatory diseases or after myocardial infarction. Among others, a small pilot series examined patients either with acute myocardial infarction or acute myocarditis with cardiac MRI and SSTR2-targeted PET/CT. Results showed a good concordance between MRI findings indicating acute myocardial damage and tracer uptake.^12^

Tarkin et al. were able to show [68Ga]Ga-DOTATATE uptake not only in patients immediately after acute myocardial infarction but also in patients with chronic ischemic heart disease.^13^ Most recently, first data from an ongoing prospective study further supports the potential for detection of prolonged macrophage activity months after the initial acute event.^14^

Regardless of these promising results, we still have not established one routinely used technique for direct visualization of myocardial inflammation. One the one hand, not all techniques are readily available at all departments, both from a technical and from a financial point of view. On the other hand, there are a lot more to establishing a routine application than the proof of principle. Regarding the first obstacle, the availability of imaging methods, [99m]Tc-Tektrotyd scintigraphy might prove a valuable addition to the portfolio of potential targeted imaging approaches. [99m]Tc-Tektrotyd has been established as alternative for detection of somatostatin receptor expression on tumorous tissues.^15^ In a very first case report 2022, a first experience with the detection of myocardial inflammation after acute anterior wall infarction was presented.^16^ Similar to the experiences with SSTR2-targeted PET, this patient showed tracer uptake in the myocardial segments matching the findings from cardiac MRI. Following this very first proof of principle, Sazonova et al. now present a further look at this imaging approach.^17^ They focused on using [99m]Tc-Tektrotyd scintigraphy in order to detect the myocardial changes caused by myocardial infarction over a time period of 6 months. Similar to previous endeavors, the case number is small but includes a well-defined homogeneous group of patients and a multimodal and serial imaging approach is chosen for characterization of the acute and chronic changes within the myocardium. Within the small patient group, the authors were able to link the [99m]Tc-Tektrotyd uptake to the extent of myocardial damage immediately after the acute event and to significantly predict changes in the end-diastolic volume after six months. With these findings, the authors fall in line with previous SSTR-targeted imaging works. However, follow-up information with the help of repeated myocardial scans with nuclear medicine techniques are rare. Sazonova et al. add to the available data with their follow-up study. Interestingly, even in this small and homogeneous group of patients, the tracer uptake at the follow-up time point shows quite a variation in results. Based on 11 patients, conclusions regarding the clinical interpretation should be drawn carefully as each sub-group consists of only few patients. Nevertheless, these few data hint at individual inflammatory responses, highlighting the need for imaging techniques that predict and detect these individual inflammatory responses.

Small-sized cases series and pilot studies such as the presented are a valuable and necessary step for transferring the principle of targeted imaging from oncology to cardiology. Following these works of proof of principle, the next challenge is to transfer the results into a wider application and to win over the clinical teams to use these imaging techniques. As a foundation, careful interpretation of technical aspects and analysis of potential pitfalls have to serve as advocates for credibility and applicability.

The target lesions often are delicate, such as atherosclerotic plaques. These lesions easily have dimensions less than 5 mm. Systematic analysis of tracer uptake and confounding factors are necessary to validate a relevant tracer uptake from potential image noise and to prevent misleading correlations with clinical markers. Furthermore, knowledge of physiological tracer uptake of different organs has to be obtained. However, true healthy reference cohorts are not available due to the exposure to ionizing radiation during the imaging procedure. Therefore, physiological distribution of SSTR2-targeted tracer uptake has been described in patients with suspicion of malign diseases but with non-pathological scans.^18^ Earlier studies with SSTR-targeted PET/CT showed that there is a strong tracer uptake in the liver. This might influence the sensitivity for inflammation in the inferior myocardial wall.^12^

Last but not least, the optimal timing for direct visualization of inflammation has yet to be determined. We know that cell activation after acute myocardial infarction follows a complex time line with different macrophage subtypes being activated at different time points.^5^ This leaves the question, whether by choosing the timing of the imaging procedure, different aspects of the inflammatory response are detected. In the studies so far, a timeframe from 2 to 11 days after the acute event were covered.^12,13,16^ As of yet, the data are too few to draw definitive conclusions, but this open question will have to be addressed in future.

In conclusion, studies like the presented are a vital step to push existing imaging techniques into new fields of applications. In doing so, a valuable key to the direct visualization of inflammation can be obtained. Nevertheless, following this first success, further challenges such as technical considerations and clinical interpretation of obtained data will have to be overcome in order to establish a routinely used application of SSTR2-targeted imaging. Once achieved, it promises to be the tool to identify patients who will benefit from targeted therapies proposed in trials, such as CANTOS.
